# Effect of candesartan monotherapy on lipid metabolism in patients with hypertension: a retrospective longitudinal survey using data from electronic medical records

**DOI:** 10.1186/1475-2840-9-38

**Published:** 2010-08-16

**Authors:** Yayoi Nishida, Yasuo Takahashi, Tomohiro Nakayama, Masayoshi Soma, Noboru Kitamura, Satoshi Asai

**Affiliations:** 1 Division of Genomic Epidemiology and Clinical Trials, Advanced Medical Research Center, Nihon University School of Medicine, 30-1 Oyaguchi-Kamimachi, Itabashi-ku, Tokyo 173-8610, Japan; 2 Division of Clinical Trial Management, Advanced Medical Research Center, Nihon University School of Medicine, 30-1 Oyaguchi-Kamimachi, Itabashi-ku, Tokyo 173-8610, Japan; 3 Division of Laboratory Medicine, Department of Pathology and Microbiology, Nihon University School of Medicine, 30-1 Oyaguchi-Kamimachi, Itabashi-ku, Tokyo 173-8610, Japan; 4 Division of General Medicine, Department of Medicine, Nihon University School of Medicine, Tokyo, 30-1 Oyaguchi-Kamimachi, Itabashi-ku, Tokyo 173-8610, Japan; 5 Division of Hematology and Rheumatology, Department of Medicine, Nihon University School of Medicine, Tokyo, 30-1 Oyaguchi-Kamimachi, Itabashi-ku, Tokyo 173-8610, Japan

## Abstract

**Background:**

Studies focusing on the add-on effects of angiotensin II type 1 receptor blockers (ARBs) other than their antihypertensive effect are receiving attention. However, the effects of prolonged administration of ARBs on lipid metabolism in clinical cases are unclear. Our aims were to survey the changes in plasma lipid profile in patients with hypertension over a one-year period, and to examine the correlations between these values and the time after the start of ARB monotherapy with candesartan.

**Methods:**

We carried out candesartan monotherapy in patients with mild to moderate hypertension and examined the longitudinal changes in plasma lipid profile. Data from 405 patients for triglyceride (TG), 440 for total cholesterol (TC), 313 for high density lipoprotein cholesterol (HDL-C) and 304 for low density lipoprotein cholesterol (LDL-C) were obtained from the electronic medical records (EMRs) in the Clinical Data Warehouse (CDW) of Nihon University School of Medicine (NUSM). The inverse probability of treatment weighting (IPTW) method (calculated from the inverse of the propensity score) was used to balance the covariates and reduce bias in each treatment duration. Linear mixed effects models were used to analyse the relationship between these longitudinal data of blood examinations and covariates of patient sex, age, diagnosis of diabetes mellitus (DM) and duration of candesartan monotherapy.

**Results:**

Plasma HDL-C level was associated with sex, duration of treatment, and interaction of sex and treatment duration, but not with age or diagnosis of DM. HDL-C level was significantly decreased during the 6~9 months period (p = 0.0218) compared with baseline. TG and TC levels were associated with sex, but not with age, diagnosis of DM or treatment duration. LDL-C level was not associated with any covariate. Analysis of the subjects divided by sex revealed a decrease in HDL-C in female subjects (during the 6~9 months period: p = 0.0054), but not in male subjects.

**Conclusions:**

Our study revealed that administration of candesartan slightly decreased HDL-C in female subjects. However, TG, TC and LDL-C levels were not influenced by candesartan monotherapy. Candesartan may be safely used for patients with hypertension with respect to lipid metabolism, because the effect of candesartan on lipids may be small.

## Background

Candesartan cilexetil is a selective angiotensin II type I receptor blocker (ARB). It is known that some ARBs improve insulin resistance [1], and we reported that monotherapy with ARBs including candesartan had a favorable effect on glucose metabolism [2]. Previous clinical trials showed that candesartan-based treatment reduced non-fatal strokes in elderly hypertensive patients [3], and that a 7-day course of candesartan after an acute ischaemic stroke significantly improved cardiovascular morbidity and mortality [4]. Pfeffer *et al*. reported that administration of candesartan to patients with chronic heart failure improved cardiovascular morbidity and mortality [5]. These large-scale clinical trials suggested the possibility that candesartan has an add-on effect to reduce cardiovascular risk. Meanwhile, an animal study showed that candesartan increased peroxisome proliferator-activated receptor-γ (PPAR-γ) mRNA expression and serum adiponectin level [6]. Therefore, it has been suggested that candesartan has a potential effect on lipid metabolism. A recent clinical study on the effect of candesartan on lipid metabolism showed that total cholesterol (TC) and low density lipoprotein cholesterol (LDL-C) levels were significantly decreased in hypertensive patients administered candesartan for at least 6 months [7]. However, the effects of prolonged administration of candesartan on lipid metabolism in patients are unclear.

In this study, we carried out candesartan monotherapy in patients, examined the longitudinal changes in plasma lipid profile up to 12 months, and studied the correlation between the profile and the duration of administration.

## Methods

### Study Population

**Figure 1:**
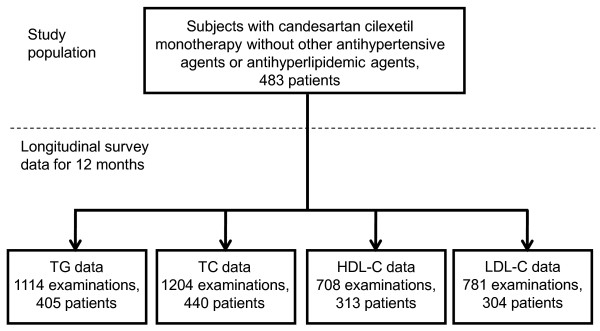
**Study population**. Medical record reviews of longitudinal survey data were carried out for 15 months; from 3 months before to 12 months after the start of candesartan monotherapy. Detailed exclusion criteria are described in the Methods.

The data for this retrospective analysis were collected from electronic medical records (EMRs) stored in the Nihon University School of Medicine (NUSM) Clinical Data Warehouse (CDW), which integrates clinical data from hospital information systems (HIS) at three hospitals affiliated to NUSM [2]. NUSM's CDW is a comprehensive data warehousing facility that provides data services to users across the clinical and research sectors of NUSM. The experimental protocol was approved by the Ethical Committee of Nihon University School of Medicine. The study subjects consisted of 483 Japanese patients with mild to moderate hypertension, aged 20 years or older who had been treated initially with candesartan cilexetil monotherapy (range: 1~12 mg/day, 93.5% of administration was in the range of 2~8 mg/day) for at least 4 weeks during the period from November 2004 to October 2009, as shown in Figure 1. Patients who had received antihyperlipidaemic agents were excluded from the study. Patients who had received other antihypertensive agents, such as an ARB other than candesartan, angiotensin-converting enzyme inhibitor (ACEI), calcium channel blocker, alpha-blocker, beta-blocker, alpha+beta-blocker, alpha-agonist or thiazide, during the 3 months before candesartan cilexetil administration were excluded from the study. In addition, patients with haemoglobin A1c (HbA1c) of 8.0% or higher were eliminated to exclude patients with very poor glycaemic control. Clinical data from the study subjects included sex, age at the start of treatment, diagnosis of diabetes mellitus (DM) according to the Committee for the Classification and Diagnosis of Diabetes Mellitus of the Japan Diabetes Society (defined as fasting plasma glucose level ≥126 mg/dl, casual plasma glucose level ≥200 mg/dl, plasma glucose 2 h after 75 g glucose load ≥200 mg/dl, or HbA1c level ≥6.5% [8]), duration of treatment, results of blood examinations including triglyceride (TG), TC, high density lipoprotein cholesterol (HDL-C) and LDL-C, which were determined at routine clinical visits, and date of examination. A total of 405 patients from this population were eligible for the study of TG, 440 for TC, 313 for HDL-C and 304 for LDL-C.

### Statistical analysis

Our main explanatory variables included sex, age at start of treatment, diagnosis of DM, and "duration" defined as the timing of measurement in days since the start of treatment as follows; baseline (within 3 months before start of treatment), 0~3 M (>0, ≤3 months), 3~6 (>3, ≤6 months), 6~9M (>6, ≤9 months) and 9~12 M (>9, ≤12 months). Our main response variables were repeated measurements of TG, TC, HDL-C and LDL-C levels in blood before and after candesartan cilexetil monotherapy. These data were not randomized, and were inherently unbalanced because the number and timing of the repeated measurements were different among individuals. The composition of the patients and the number of examinations were not equal for each of the treatment durations, and may have been time-dependently affected by candesartan monotherapy itself. Marginal structural models using inverse probability of treatment weighting (IPTW) have been recently developed to solve this problem that the treatment effects are affected by time-dependent confounders that are themselves affected by the treatment [9]. Therefore, we used IPTW to balance the treatment durations so as to reduce bias in the patient background and obtain a better idea of the effect of treatment on the outcome of compliance. IPTW is calculated as the inverse of the propensity score. The propensity score method introduced by Rosenbaum and Rubin is an effective tool to reduce bias in nonrandomized studies including unbalanced data [10]. The traditional propensity score method such as matching, stratification and covariance adjustment is mostly used in binary-value treatment [11-13]. Recently, there have been many reports trying to expand its application to more than two treatments [14-16]. We referred to the method introduced by Leslie et al., and used an IPTW-linear mixed effect model to reduce bias in the duration of treatment [15-18]. This method consisted of three steps as follows. As the first step, we used propensity score adjustment to account for potential selection bias in each treatment duration. We used a logistic regression model to calculate the propensity score as the probability of examinations with each treatment duration. The variables in this step included sex, age and diagnosis of DM. As the second step, IPTW was calculated as the inverse of the propensity score. By using this IPTW in the next step, bias in each treatment duration could be minimized. To give more weight to smaller treatment groups, a weight was created that reflects the sample size for each blood examination. As the third step, an IPTW-linear mixed effect model was fitted to analyse the relationship between these longitudinal data of blood examinations and all other covariates. We fitted repeated measurement analysis (covariance structure: Compound Symmetry) to the data, including sex, age, diagnosis of DM and a confounding factor of interaction of sex and duration of treatment as fixed effect covariates, and duration of treatment as a repeated effect covariate. We selected the Kenward-Roger method to compute the denominator degrees of freedom for the tests of fixed effects. A multiple-comparison test (Dunnett-Hsu post-hoc analysis) was used to compare the differences in means between "baseline" as a reference and other treatment duration periods. A result was considered statistically significant if the p value was less than 0.05. All statistical analysis was performed with SAS 9.1.3 (SAS Institute Inc., Cary, NC) statistical software.

## Results

### Characteristics of study sample

Of the study population of 483 patients with hypertension, those who had been treated with candesartan cilexetil monotherapy but no antihyperlipidaemic agent were subjected to statistical analysis (Figure 1). The numbers of blood examinations were as follows; TG: total 1114 examinations (405 patients with 2.75 measurements per patient), TC: total 1204 examinations (440 patients with 2.74 measurements per patient), HDL-C: total 708 examinations (313 patients with 2.26 measurements per patient), LDL-C: total 781 examinations (304 patients with 2.57 measurements per patient) (Figure 1). Table 1 shows the details of patient information and data. Approximately 36% of patients who underwent blood examination were female and 64% were male. Approximately 47% of patients had DM. Mean age was approximately 61 years, and the age range was 20 to 91 years.

**Table 1:** Frequency distribution of blood examination data.

Variables	TG	TC	HDL-C	LDL-C
**Patient information**				
Number of patients	405	440	313	304
Age, years				
Mean ± SE	61.2 ± 14.5	61.7 ± 14.9	60.3 ± 14.6	60.2 ± 14.4
Range	20 - 86	20 - 91	20 - 86	20 - 86
Sex, number (%)				
Female	142 (35.1)	165 (37.5)	113 (36.1)	102 (33.6)
Male	263 (64.9)	275 (62.5)	200 (63.9)	202 (66.4)
DM, number (%)				
No	217 (53.6)	252 (57.3)	160 (51.1)	151 (49.7)
Yes	188 (46.4)	188 (42.7)	153 (48.9)	153 (50.3)
**Data information (number of examinations)**				
Treatment duration				
Baseline	464	504	312	298
0~3M	221	262	137	156
3~6M	185	190	114	136
6~9M	136	140	84	107
9~12M	108	108	61	84

TG: triglyceride, TC: total cholesterol, HDL-C: high density lipoprotein cholesterol, LDL-C: low density lipoprotein cholesterol, DM: diagnosis of diabetes mellitus.

### Relationship of covariates to plasma lipid profile

Table 2 shows the results of Type III test of propensity score-weighted linear mixed effect models fitted to plasma TG, TC, HDL-C and LDL-C data. There was a significant association between HDL-C level and sex (p = 0.0017), treatment duration (p = 0.0427), and interaction of sex and treatment duration (p = 0.0349), but no association between HDL-C and age or diagnosis of DM. There was a significant association between TG and sex (p = 0.0041), but no association between TG and age, diagnosis of DM, treatment duration or interaction of sex and treatment duration. There was a significant association between TC and sex (p = 0.0032), but no association between TC and age, diagnosis of DM, treatment duration or interaction of sex and treatment duration. There was no association between LDL-C and sex, age, diagnosis of DM, treatment duration or interaction of sex and treatment duration. Candesartan monotherapy was associated with a decrease in HDL-C level, but had little effect on other lipid parameters.

**Table 2:** Relationship of covariates to plasma lipid profile

**Effect**	**DF**	**TG**		**TC**		**HDL-C**		**LDL-C**	
				
		**F-value**	**p value**	**F-value**	**p value**	**F-value**	**p value**	**F-value**	**p value**
				
Sex	1	8.32	0.0041*	8.74	0.0032*	9.99	0.0017*	2.04	0.1539
DM	1	0	0.9978	1.42	0.2333	1.69	0.1949	0.34	0.5628
Age	1	1.79	0.1819	0.95	0.3311	0.04	0.8429	0.24	0.627
Treatment duration	4	0.4	0.812	0.68	0.6086	2.49	0.0427*	0.44	0.7812
Sex*Treatment duration	4	1.28	0.278	1.12	0.3434	2.61	0.0349*	1.82	0.1243

DM: diagnosis of diabetes mellitus, Sex*Treatment duration: interaction of sex and duration of treatment, TG: triglyceride, TC: total cholesterol, HDL-C: high density lipoprotein cholesterol, LDL-C: low density lipoprotein cholesterol, DF: degrees of freedom, p value: p value of covariate, *: p < 0.05.

Table 3 shows the results of Dunnett's multiple-comparison test of propensity score-weighted linear mixed effect models fitted to plasma TG, TC, HDL-C and LDL-C data. HDL-C was slightly but significantly decreased in the '6~9M' period compared with baseline (1.40 vs. 1.47 nmol/L, p = 0.0218), but was not significantly changed in the other periods, '0~3M', '3~6M' and '9~12M', compared with baseline. There was no significant change in TG, TC or LDL-C in any treatment duration period.

**Table 3:** Multiple comparison test of levels of lipid parameters among treatment duration periods.

Treatment duration	TG (nmol/L)			TC (nmol/L)		
		
	LS mean ± SE	95% CI	p value	LS mean ± SE	95% CI	p value
		
Baseline	1.51 ± 0.05	1.42/1.61	reference	5.30 ± 0.04	5.21/5.39	reference
0~3M	1.52 ± 0.07	1.39/1.65	1	5.24 ± 0.05	5.14/5.35	0.5876
3~6M	1.53 ± 0.07	1.39/1.67	0.9984	5.30 ± 0.06	5.19/5.42	1
6~9M	1.61 ± 0.08	1.45/1.77	0.6205	5.26 ± 0.06	5.13/5.39	0.9223
9~12M	1.54 ± 0.08	1.36/1.72	0.9963	5.34 ± 0.07	5.20/5.48	0.9717
						

**Treatment duration**	**HDL-C (nmol/L)**			**LDL-C (nmol/L)**		
		
	**LS mean ± SE**	**95% CI**	**p value**	**LS mean ± SE**	**95% CI**	**p value**
		
Baseline	1.47 ± 0.02	1.42/1.52	reference	3.12 ± 0.05	3.02/3.21	reference
0~3M	1.45 ± 0.03	1.40/1.51	0.858	3.10 ± 0.05	2.99/3.21	0.9917
3~6M	1.45 ± 0.03	1.40/1.51	0.8875	3.05 ± 0.06	2.93/3.16	0.5653
6~9M	1.40 ± 0.03	1.34/1.46	0.0218*	3.11 ± 0.06	2.98/3.23	0.9999
9~12M	1.41 ± 0.03	1.34/1.47	0.1247	3.08 ± 0.07	2.95/3.22	0.9778

TG: triglyceride, TC: total cholesterol, HDL-C: high density lipoprotein cholesterol, LDL-C: low density lipoprotein cholesterol, LS mean: least squares mean, SE: standard error, CI: confidence interval, p value: p value of treatment duration (compared with baseline, multiple-comparison test: Dunnett-Hsu post-hoc analysis), *: p < 0.05.

**Figure 2:**
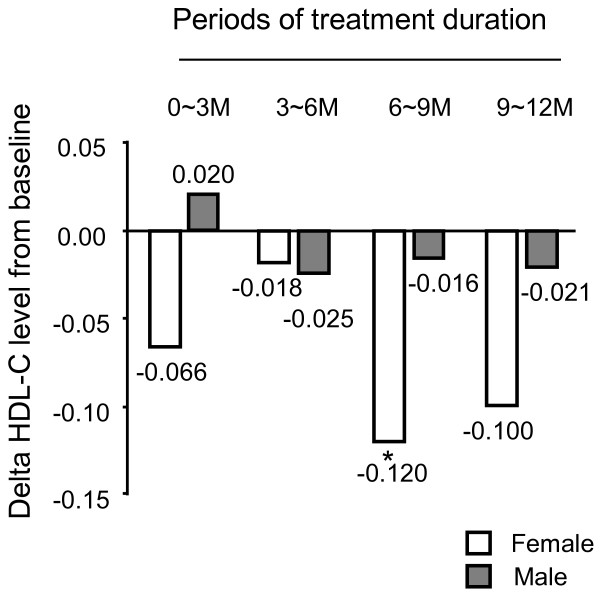
**Mean change in HDL-C level in each treatment duration period from baseline**. White squares show female results and black squares show male results. *: p < 0.05.

We further analysed the data divided by sex, because HDL-C was also significantly associated with sex and interaction of sex and treatment duration. Figure 2 shows the change in HDL-C level in each treatment duration period, compared with baseline. HDL-C was significantly decreased in the '6~9M' period compared with baseline in female data (1.45 vs. 1.57 nmol/L, p = 0.0054), but was not significantly changed in male data (Figure 2 and Table 4). Candesartan monotherapy had an unfavorable effect on HDL-C in female subjects, but HDL-C level in female subjects was within the normal range during the study period.

**Table 4:** Multiple comparison test of HDL-C level among treatment duration periods, by sex.

**Treatment duration**	**Female HDL-C (nmol/L)**				**Male HDL-C (nmol/L)**			
		
	**Exam.N**	**LS mean ± SE**	**95% CI**	**p value**	**Exam.N**	**LS mean ± SE**	**95% CI**	**p value**
	
Baseline	102	1.57 ± 0.04	1.50/1.65	reference	210	1.38 ± 0.04	1.33/1.44	reference
0~3M	53	1.51 ± 0.04	1.42/1.59	0.1087	84	1.40 ± 0.03	1.34/1.47	0.889
3~6M	41	1.56 ± 0.04	1.47/1.64	0.9579	73	1.36 ± 0.03	1.29/1.42	0.8245
6~9M	26	1.45 ± 0.05	1.36/1.55	0.0054*	58	1.37 ± 0.04	1.30/1.44	0.9719
9~12M	15	1.47 ± 0.06	1.36/1.59	0.1423	46	1.36 ± 0.04	1.28/1.44	0.9444

HDL-C: high density lipoprotein cholesterol, Exam N: number of examinations, LS mean: least squares mean, SE: standard error, CI: confidence interval, p value: p value of treatment duration period (compared with baseline, multiple-comparison test: Dunnett-Hsu post-hoc analysis), *: p < 0.05.

## Discussion

In this study, we examined the changes in laboratory data of lipid metabolism up to 12 months in patients receiving candesartan monotherapy. Because this survey was a longitudinal study, the study population represented its own time-related control group. In this retrospective longitudinal survey, we found a significant reduction in HDL-C level from the start to 6~9 months of candesartan administration (Tables 2 and 3). These results suggest that candesartan has an unfavorable effect on lipid metabolism, with a reduction in HDL-C level by administration of candesartan for 6~9 months in female subjects. However, the effect of candesartan to reduce HDL-C level was transient and limited to female subjects. Moreover, HDL-C level was within the normal range throughout the study period (Table 4). Therefore, this slightly unfavorable effect of candesartan monotherapy on HDL-C in female subjects may not be a problem in clinical practice. Regarding lipid metabolism, candesartan may be safely used for patients with hypertension in the long term up to 12 months. Bramlage et al. reported that ARBs provide substantial cost savings and may prevent cardiovascular morbidity and mortality based on more complete antihypertensive coverage, suggesting that ARBs are an attractive choice for long-term treatment of hypertension [19]. Our findings on the safety of long-term use of candesartan with respect to lipid metabolism reinforce these benefits of ARBs.

Only a few studies have examined the effect of candesartan on lipid metabolism; however, some showed that administration of candesartan had no effect on lipid metabolism. When HDL-C, TC, and TG levels were compared between before administration and after 2 weeks of candesartan administration to patients with essential hypertension, no significant difference was found [20]. Also, when HDL-C, LDL-C, TC, and TG levels were compared between before administration and after 8 weeks of candesartan administration to patients with mild hypertension and type 2 DM, no significant difference was found [21]. When HDL-C, LDL-C, TC, and TG levels were compared between before administration and after 12 weeks of candesartan administration to patients with mild hypertension and type 2 DM, no significant difference was found [22]. Furthermore, when HDL-C, LDL-C, TC, and TG levels were compared between before administration and after 12 months of candesartan administration to patients with mild hypertension and type 2 DM, no significant difference was found [23]. Supporting these previous reports, no significant changes were observed with candesartan administration for less than 6 months and more than 9 months in our study.

A close relationship has been suggested between lipid and glucose metabolism, but there was no association between lipid metabolism and the covariate of DM in our study results (Table 2). The reason for this may be that only patients with well-controlled blood glucose were selected during the subject selection stage in this study, because patients with a very high HbA1c level (≥8.0%) were excluded. As a result, there was a smaller effect of glucose metabolism abnormality on lipid metabolism.

In our study, HDL-C level was significantly reduced in female subjects, but not in male subjects (Figure 2 and Table 4). The reason for this discrepancy may be as follows. First, the effect of candesartan on HDL-C may be stronger in patients with a high HDL-C level than in those with a low HDL-C level. It is well known that there is a sex-difference in plasma lipid profile; plasma HDL-C level is generally higher in female subjects than in male subjects [24,25]. Second, the effect of candesartan on HDL-C may reflect its effects on hormones. A previous report revealed that oestrogen increases HDL-cholesterol [26]. However, the reason for this discrepancy between male and female subjects is still unclear.

This study was a retrospective database study, which can provide many benefits as follows: First, real-time data can be provided quickly and cost-effectively. Second, the sample sizes are relatively large. Third, the influences on the patients' risk are minimal. These strengths readily led to stimulating studies and promising outcomes [27]. On the other hand, our study was a retrospective observational study, which has some limitations with respect to the potential for selection bias and confounding factors. However, these problems caused by non-randomized data could be solved by combination with robust statistics; for example, propensity score method [9]. Our study, with appropriate application of statistical analysis techniques; i.e., the propensity score adjustment and weighted-linear mixed model, may yield findings with validity, and help physicians make decisions on drug selection.

## Conclusions

In this study, we examined the effect of candesartan monotherapy on lipid metabolism. Our results revealed that HDL-C level in female subjects declined from 6 to 9 months after the initiation of candesartan monotherapy. However, the reduction of HDL-C level was transient and was observed only in female subjects. Moreover, the HDL-C level was within the normal range throughout the study period. In addition, TG, TC and LDL-C levels were not influenced by candesartan monotherapy. These results indicate a lack of obvious evidence showing an unfavorable influence of candesartan on lipid metabolism. Therefore, the influence of candesartan monotherapy on lipid metabolism may be small and may not be a clinical problem. In the field of lipid metabolism, candesartan may be safely used for patients with hypertension.

## Competing interests

The authors declare that they have no competing interests.

## Authors' contributions

SA and YN conceived the study and participated in its design. YT performed the statistical analyses. TN, NK and MS drafted the manuscript and interpreted the data. All authors have read and approved the final manuscript.
